# Inter-rater reliability for movement pattern analysis (MPA): measuring patterning of behaviors versus discrete behavior counts as indicators of decision-making style

**DOI:** 10.3389/fpsyg.2014.00605

**Published:** 2014-06-20

**Authors:** Brenda L. Connors, Richard Rende, Timothy J. Colton

**Affiliations:** ^1^Chief of Naval Operations Strategic Studies Group, Naval War CollegeNewport, RI, USA; ^2^Social Behavioral Research ApplicationsSouth Dartmouth, MA, USA; ^3^Department of Government, Harvard UniversityCambridge, MA, USA

**Keywords:** decision-making, individual differences, observational methods, movement pattern analysis, inter-rater reliability

## Abstract

The unique yield of collecting observational data on human movement has received increasing attention in a number of domains, including the study of decision-making style. As such, interest has grown in the nuances of core methodological issues, including the best ways of assessing inter-rater reliability. In this paper we focus on one key topic – the distinction between establishing reliability for the patterning of behaviors as opposed to the computation of raw counts – and suggest that reliability for each be compared empirically rather than determined *a priori*. We illustrate by assessing inter-rater reliability for key outcome measures derived from movement pattern analysis (MPA), an observational methodology that records body movements as indicators of decision-making style with demonstrated predictive validity. While reliability ranged from moderate to good for raw counts of behaviors reflecting each of two Overall Factors generated within MPA (Assertion and Perspective), inter-rater reliability for patterning (proportional indicators of each factor) was significantly higher and excellent (ICC = 0.89). Furthermore, patterning, as compared to raw counts, provided better prediction of observable decision-making process assessed in the laboratory. These analyses support the utility of using an empirical approach to inform the consideration of measuring patterning versus discrete behavioral counts of behaviors when determining inter-rater reliability of observable behavior. They also speak to the substantial reliability that may be achieved via application of theoretically grounded observational systems such as MPA that reveal thinking and action motivations via visible movement patterns.

## INTRODUCTION

Experienced leaders can vary greatly in their decision-making style (Connors, 2006, unpublished). Such variation can be observed in various workplace contexts. For example, military leaders can differ greatly across dimensions of decision-making style such as rational, avoidant, spontaneous, and dependent (Thunholm, 2004, 2009). The unique leadership styles of world leaders are evident on the world stage and can be observed in real time and deciphered via analysis of videotape interaction and comparative analysis of different leaders who, for example, show differences in their inclination to implement action (Connors, 2006, unpublished). Individuals in professional capacities that demand decision-making expertise – such as local, state, and central government leaders – differ with respect to their need for cognition when faced with decision-making tasks (Carnevale et al., 2010). Given the recognized need for appreciating and assessing individual differences in decision-making style (Mohammed and Schwall, 2009; Del Missier et al., 2010; Weber and Morris, 2010; Appelt et al., 2011; Harman, 2011; Bruine de Bruin et al., 2012), a challenge for research is to establish methods that can be used reliably to gain insight into the range of decision-making propensities in those charged with making important decisions on a regular basis.

While methods designed to capture these important individual variations in decision-making style have depended mostly upon self-report, there is increasing attention to the unique advantages of observational methods for deciphering telling indicators of decision-making style. This includes indicators that focus on movement as a window into cognition, including the processes by which individuals arrive cognitively at taking action during the decision-making process (see Connors et al., 2013). Of particular salience is the ecological validity of observational methods that capture behaviors of interest in real time and real settings, and the potential to derive from observable behaviors unique insights in individual differences in cognitive and behavioral styles (Baumeister et al., 2007; Furr, 2009a).

It is obvious that in order for observational methodologies to be valuable research tools, they need to be examined with respect to core psychometric properties, including inter-rater reliability. Inter-rater reliability is a critical consideration for observational systems that rely on the ability of trained coders to converge on their detection of key behavioral indicators comprising the coding system. Indeed, one argument for the utility of observational research is that the establishment of inter-rater reliability provides evidence of a more “objective” data collection process that avoids the pitfall of rater bias that can impact perceptual ratings typically gathered via self-report. This can be seen by a proliferation of papers focused on measuring inter-rater reliability for a variety of observational coding systems that measure movement (e.g., Ebersol and Armstrong, 2006; Bao et al., 2009; Kociolek and Keir, 2010; Xu et al., 2011; Bussman and van den Berg-Emons, 2013).

While the determination of inter-rater reliability may seem straightforward, many considerations go into making the calculation maximally informative. It has been suggested that many core principles of, and techniques for, establishing inter-rater reliability of observational methods need to be revisited, as the nuances of these issues are not always explored in sufficient depth (Haidet et al., 2009; Hallgren, 2012). In this paper, we take on one fundamental consideration that is underrepresented in the literature – the distinction between establishing reliability for the *patterning* of behavior within subjects versus computation based on *raw counts*. This issue is distinct from considerations of absolute agreement versus consistency in ratings, which has been well articulated in the literature (see Hallgren, 2012). While many researchers are more concerned with consistency across raters than with absolute agreement – such that inter-rater reliability can be achieved in the relative and not absolute sense – some observational systems are more concerned with detecting patterns of behaviors within each subject, and the variation in patterning across subjects. In these cases, it may be argued that while subjects may differ greatly in their raw counts of target behaviors, this is not the most telling metric in prediction models. Rather, the proportion of behaviors, relative to the total raw count for each subject, is of most interest. Coders may be attuned to the patterning of behaviors within subjects, and may achieve high levels of reliability on those patterns, with the aim of best predicting individual differences across subjects. In this paper, we illustrate these considerations via computation of inter-rater reliability for key outcome measures derived from movement pattern analysis (MPA), an observational methodology that records body movements as indicators of decision-making style with demonstrated predictive validity (Connors et al., 2013).

### INTER-RATER RELIABILITY AND MPA

Movement pattern analysis is a theoretically based observational methodology that objectively codes specific body movements to provide indicators of decision-making style. When applied to a group of individuals, it provides a contrastive analysis of how they vary in the manner in which they approach decision-making. It has been used with regularity for over five decades in the business world to inform executive selection and the building of management teams (see Moore, 2005; Lamb, 2012) and has been applied to provide insight into the decision-making style of military and world leaders (Connors, 2006, unpublished; Connors et al., 2013). The MPA decision framework assesses body movements corresponding to the whole decision process of the individual including multiple cognitive stages as well as the motivational degree to which an individual will actually undertake decision implementation. While everyone engages all stages while faced with decision-making tasks and opportunities, the premise is that individuals vary in the way they prioritize and sequence some processes over others. Such differences in individual patterning in movements that support and reflect different stages of processing define decision-making style in the MPA model. Connors et al. (2013) provided data supporting the predictive validity of MPA, using observable indicators of decision-making behavior in a laboratory setting.

### EVENT-BASED CODING OF MOVEMENT

At the core of MPA is the coding, typically done from videotaped behavior, of “posture-gesture mergers” (PGMs), which are observable events in which a posture (movement consistent as a whole throughout the body) becomes integrated with a gesture (a movement confined to a part or parts; Lamb, 2012). Connors et al. (2013) have provided an overview of MPA, including detailed examples of a number of PGMs that are recorded using this methodology. The MPA approach requires each observer to record raw counts of PGMs within twelve categories of decision-making process (referred to as Action Motivations). These can helpfully be aggregated into two Overall Factors of Assertion and Perspective^1^. As summarized in Connors et al. (2013), MPA posits that individuals need to balance their actions/motivations between exerting tangible energy in the environment that *get results* (Assertion), versus those motivations that shape the body to position the person to *receive from the environment input to initiate or create the result* (Perspective). Differences in how individuals achieve their own *balance* between the complementary processes of Assertion and Perspective are proposed to capture different decision-making styles.

Recording PGMs is an example of “event-based coding” – there is neither a time window imposed (such as making a rating every 30 s or every minute) nor a “yes/no” checklist to follow (such as saying “check yes if you observed a PGM in a given category over the last minute”). Event-based coding systems are complex to apply because they are open-ended and unfold in real chronological time. It can thus be a challenge to determine the best method for establishing inter-rater agreement. Recording time-locking responses across coders would be one possibility, though this would be most appropriate only if the coding system generated summary data at this level of specificity. As emphasized in recent thinking on observational methodology, estimates of inter-rater reliability must focus on the deliverable outcome measures used in prediction models (Haidet et al., 2009; Hallgren, 2012).

With this point in mind, it’s important to consider that MPA, like many observational coding systems, generates percentage-based scores. They are believed to be more reliable and more to the point of the coding, as the idea is to determine how a given individual’s pattern of behaviors is distributed across different codes (which, in this case, represent proportional allocation of total decision-making motivation to different stages of the decision-making process). Here each person is considered, behaviorally, to be their own “denominator” in the sense that their patterning is determined relative to their own baseline of behavior. Theoretically, it is the *patterning* of PGMs across different stages of decision-making process that defines individual differences in decision-making style – not differences in *total counts* of PGMs within the different stages. Movement analysis provides both a quantitative and qualitative capture by focusing overtly on the structure and dynamics of the pattern of the motion within the stream of real-time behavior (Moore, 2005; Lamb, 2012). In MPA, an expert coder is trained to assess how an individual balances the various types of decision-making processes in real time. In this sense, there is no valence attached to how a person achieves such a balance, but rather the goal is to reveal their motivational tilt via observed movement. Thus, the calculation of percentage data not only makes sense from the perspective of observational methodology, but also dovetails with the theoretical premise of MPA, as the human body reflects consistency of pattern through the seminal phenomena of PGMs.

The idea that MPA captures a stable pattern of how each individual balances different aspects of the decision-making process also resonates with a broader interest in capturing individual-level consistencies in personality research, such as methods used for profile analysis. For example, Furr (2009b) describes a methodological framework for using profile analysis to capture the replicable patterns of personality individuals bring to multiple situations. As articulated by Furr (2009b), by using methods to quantify such behavioral consistency across situations, the profile approach integrates an idiographic profile with the nomothetic approach – similar conceptually to what MPA can achieve. Thus, while our approach is rather specific to the MPA coding system – as the patterning is defined here as an individually expressed balance between the two Overall Factors – there is, as noted above, a conceptual similarity to recent trends in personality research, including innovative methods to examine the replicability of a pattern of correlations that may reflect stability of behavioral indicators (Sherman and Wood, 2014).

Prior research has suggested high inter-rater reliability for the MPA coding system, focusing specifically on calculations using percentage-based data (Winter et al., 1989; Winter, 1992). We recently confirmed high reliability for the Overall Factors of Assertion and Perspective. That said, given the recent calls in the literature to address core issues in evaluating inter-rater reliability for observational methods, the distinction between examining raw counts of PGMs by different raters and the patterning of responses as recorded by different raters should be viewed, in significant part, as an empirical matter. To that end, in this paper we present computations of both forms of inter-rater reliability in a study that conducted MPA evaluations of military leaders (see Connors et al., 2013). Given the conceptual approach of MPA, we hypothesize that inter-rater reliability for patterning should be superior to that calculated for raw counts. In addition, as reliability should, to a degree, correlate with predictive validity, we also conduct analyses to gage the unique prediction of each with respect to decision-making behavior recorded in a hypothetical decision-making task (see Connors et al., 2013), given the importance of examining the extent to which any trait-based approach predicts observable behavior (Brown and Sherman, 2014).

## MATERIALS AND METHODS

### SUBJECTS

As described in Connors et al. (2013), we recruited twelve current or retired U.S. military officers who had between 20 and 30 years of military service each. The officers represented all branches of the armed forces, including the Coast Guard, other than the Army. There were nine males and three females in the group. All subjects provided informed consent in accordance with a protocol approved by the appropriate institutional review board (IRB).

### MOVEMENT PATTERN ANALYSIS

All subjects participated in a videotaped 2-h interview with one MPA analyst/interviewer (see Connors et al., 2013) under the overall project direction of the first author, who is an Advanced MPA practitioner and Laban Certified Movement Analyst (CMA). This interview consisted of a series of open-ended questions focused on life, career history, and present situation. These provide a semi-naturalistic opportunity to observe movements posited in the MPA model to be indicators of decision-making style. As mentioned in the Introduction, the key behavioral indicator coded throughout the 2-h interview is the PGM, which is expressed in various ways during each of multiple stages (Attention, Intention, and Commitment) of the decision-making process (Moore, 2005; Lamb, 2012). More details about (and examples of) these stages, represented and coded in the MPA model as six Action Motivation behaviors (Investigating, Exploring, Determining, Evaluating, Timing, Anticipating), are presented in Connors et al. (2013). These six Action Motivation behaviors are then summed up as the two Overall Factors in the MPA model – Assertion and Perspective.

Individual differences come into play as individuals find their own balance between the complementary factors of Assertion and Perspective. Raters are required in essence to code the relative allocation of PGMs across these Overall Factors (through coding of twelve movement measures grouped into two groups of six Action Motivation behaviors indicating either Assertion or Perspective). Each individual’s decision-making style is recorded in percentage terms – the percentage of PGMs reflecting Assertion and the percentage of PGMs reflecting Perspective. As these are complementary percentages (by definition, they sum to 100%), we create a Perspective/Assertion Balance score as follows:

P/A Balance Score = %PGMs(Perspective) –%PGMs(Assertion)

This P/A Balance Score provides an overall picture of each person’s decision-making style (see Connors et al., 2013). A score of “0” reflects an individual who allocates equally to Assertion and Perspective; a positive number reflects more distribution to Perspective; and a negative number reflects more distribution to Assertion.

While prior research (Winter et al., 1989; Winter, 1992) has shown that Assertion and Perspective can be recorded with high inter-rater reliability – including our initial assessments based on a subset of subjects in this study (Connors et al., 2013) – no systematic comparisons of the inter-rater reliability of these percentage-based codes versus raw counts have been conducted. To that end, two raters coded the MPA interview for each of the twelve subjects in this study, and computed both the raw counts of PGMs in Assertion, and Perspective, respectively, along with percentage-based codes, for each coder. Three coders were used, with only two per subject contributing data for reliability analysis. Given the need to utilize outcome measures that would be used in prediction models when assessing inter-rater reliability (Hallgren, 2012), for each rater we computed two scores per subject: the P/A Balance score (described above as a percentage-based measure) and a comparable difference score based on the difference between the number (raw count) of Perspective PGMs and the number (raw count) of Assertion PGMs:

P/A Difference score = PGMs(Perspective) – PGMs(Assertion)

These two variables – P/A Balance, and P/A Difference – were used to generate different indicators of inter-rater reliability.

### HYPOTHETICAL DECISION-MAKING SCENARIOS

As described in Connors et al. (2013), subjects were presented with four hypothetical decision-making tasks (Financial, Health, Voting, and Strategy) in a laboratory setting. Subjects were given options to seek out, one at a time, additional pieces of information to consider before coming to a decision. In this context such an indicator of information search, along with the amount of chronological response time, are presumed to be sensitive quantitative indicators of decision-making process that would show differences across individuals. The number of information draws (each request for additional information) was recorded electronically for each scenario, as was response time (chronological time measured in seconds). This yielded two outcome measures of individual differences in decision-making behavior: Total Info Draws (the total number of requests for additional information summed across all four hypothetical scenarios) and Total Response Time (the total chronological time in seconds summed across all four hypothetical scenarios). Our prior work has demonstrated that the P/A Balance score is a robust predictor of both outcome measures, with a propensity for Perspective (relative to Assertion) being associated with higher Total Info Draws, and longer Total Response Time (Connors et al., 2013). We note that although these outcome measures show covariation in this sample (*r* = 0.54, *p* < 0.10), we include both in analyses as we have done previously (see Connors et al., 2013, for further discussion).

### ANALYTIC PLAN

We address two issues in the Results section. First, we generate estimates of inter-rater reliability for the P/A Balance (patterning) and P/A Difference (raw count) measures, including 95% confidence intervals (CIs) for comparison. Second, we utilize both the P/A Balance and P/A Difference measures in a stepwise regression model predicting Total Info Draws, and Total Response Time, respectively, to gain insight into the predictive power of each.

## RESULTS

### DESCRIPTIVE ANALYSES OF PREDICTORS AND OUTCOME MEASURES

The P/A Balance and P/A Difference scores correlated at 0.57 (*p* < 0.10), suggesting that while associated, they can be considered as potentially different indicators.

The average number of PGMs recorded for each subject was 155.83 (SD = 55.95), and the range was from 62 to 220. Descriptive statistics for the predictor and outcome variables are provided in **Table 1**.

**Table 1:** Descriptive statistics for predictor and outcome variables.

Variable	Mean (SD)
P/A Balance	-22.67 (22.81)
P/A Difference	-33.75 (31.81)
Total Response Time	640.70 (192.99)
Total Info Draws	16.08 (3.68)

### INTER-RATER RELIABILITY

As we were not concerned with specific rater effects – and as all raters did not rate every subject – a one-way random effects model was used (IBM SPSS Statistics 22) to calculate the intraclass correlation (ICC) for both P/A Balance and P/A Difference. **Table 2** presents the ICC, along with a 95% CI, for each measure.

**Table 2:** Intraclass correlation (ICC) coefficients and 95% confidence intervals (CIs) for patterning (P/A Balance) and raw count (P/A Difference) measures.

Measure	ICC	CI (95%)
P/A Balance	0.89	**0.77–0.95
P/A Difference	0.41	*0.02–0.69

*p < 0.05; **p < 0.01.

The most direct comparison of the ICCs in **Table 2** is offered by a consideration of the 95% CIs. These CIs do not overlap, suggesting a significant difference in the magnitude of the CIs – with the CI for P/A Balance (patterning) being significantly higher than the CI for P/A Difference (raw count). It is also noted that the ICC for P/A Balance is excellent, consistent with prior research (Winter et al., 1989; Winter, 1992).

### REGRESSION MODELS

The ICC analysis suggests that the P/A Balance score has superior inter-rater reliability, and so would be a stronger predictor of decision-making behavior as compared to a P/A Difference score. Correlational analyses provide partial support to that proposition. P/A Balance correlated 0.61 (*p* < 0.05) with Total Response Time and 0.50 (*p* < 0.10) with Total Info Draws; P/A Difference correlated 0.26 (ns) with Total Response Time and 0.47 (ns) with Total Info Draws.

A stronger test is to consider both P/A Balance and P/A Difference as potential predictors of the two outcome measures. The stepwise regression model for Total Response Time selected P/A Balance, which was significant (*t* = 2.37, *p* < 0.05), and excluded P/A Difference (*t* = 0.91, ns). For Total Info Draws, the stepwise model did not select either predictor (consistent with the pattern of correlations noted above).

## DISCUSSION

This exercise in assessing the inter-rater reliability of different representations of indicators of decision-making style is cast within a broader methodological concern with ensuring the reliability of observational techniques in behavioral research (Hallgren, 2012). While assessment of movement offers powerful insight into many dimensions of human behavior (Moore, 2005; Lamb, 2012), including the decision-making styles of world leaders (Connors, 2006, unpublished), it comes with it the burden of establishing reliability for coding systems that can be extremely complex and may present nuances not always brought to the surface when assessing inter-rater reliability (Haidet et al., 2009; Hallgren, 2012).

Our focus on MPA offers an opportunity to delve into one of the issues that can arise when working with observational data, namely the utility of raw behavioral counts versus the coding of meaningful patterning of behavior. The MPA model conceptually posits that a person’s patterning – for example their individual proclivity for balancing the demands of Perspective and Assertion across the stages of decision-making – is the most telling indicator of their decision-making style. MPA has been applied for decades in various professional contexts (particularly organizational behavior and dance) and has always emphasized this aspect of capturing the balancing of movement as an indicator of how individuals uniquely navigate the various stages in the decision-making process (Moore, 2005; Lamb, 2012). It provides a rich model and coding system for illuminating the conceptual distinction between gaging reliability for patterning of movement versus raw tallies of movement behavior.

In this sense, differences in the absolute raw counts of behavioral indicators of Assertion and Perspective – coded as tallies of PGMs that are reflective of each process – do not carry as much information as the way in which they are balanced in an individual. Put another way, even though one individual may be observed to exhibit more PGMs reflective of Perspective than another individual, such information is not as meaningful as knowing their proportional reliance on Perspective versus Assertion. Individuals provide their own baseline with respect to their total number of PGMs, and what is most revealing with respect to individual differences is the relative patterning or balance of Assertion and Perspective within each individual.

The calculation of ICCs in this study supports this idea. Coders achieved an excellent level of inter-rater reliability for the P/A Balance score. Furthermore, the reliability estimate was significantly higher than that achieved when we examined raw counts of PGMs. Two related points are important here. First, the premise of the MPA model – that raters can reliably decipher the patterning in an individual’s body movements that align to theoretical components of decision-making style – is confirmed, as it has been in prior research (Winter et al., 1989; Winter, 1992). Second, while inter-rater reliability models often permit relative differences in raw counts across raters, the idea of patterning is different, when appropriate. It is one example of bringing a more fine-tuned perspective to assessing inter-rater reliability for observational methods (see Haidet et al., 2009; Hallgren, 2012).

In addition to the inter-rater reliability analyses, we also included a multiple regression model to round out our conclusions about patterning versus raw counts. While our prior work demonstrated the strong predictive value of the P/A Balance score (Connors et al., 2013), it is important to consider it in the context of raw counts. Here our reliability analysis is consistent with prediction models, as the P/A Balance carried the predictive power in the regression model for Total Response Time. While it is assumed that the more reliable indicator would be more predictive, again our point is to put these assumptions to the test empirically. To that end, we also note that the regression model did not achieve significance for Total Info Draws. While there could be a number of methodological reasons for this (e.g., P/A Balance and P/A Difference had similar correlations with Total Info Draws, both of which did not achieve significance with the present sample size), the finding speaks to the importance of determining empirically the predictive value of patterning versus raw counts for a number of potential outcome variables. Future work with larger samples may be able to further address the associations between the measures used in this study.

Overall, there is substantial appreciation in multiple literatures on the need for nuanced analysis of inter-rater reliability when working with observational methods in general, and movement based systems in particular. Observational systems can produce a variety of measures and it is not a straightforward process to determine the most salient indicators for research. For example, the assessment of “physical activity” can yield a host of constructs and a wide variety of measurement approaches (Bussman and van den Berg-Emons, 2013). Conducting careful assessments of the inter-rater reliability of multiple ways of generating outcome measures is an essential step when sorting through, and selecting, the most telling measures for observational research, particularly when we are dealing with the complexities of recording human movement and interpreting behavior of individuals, including those in leadership positions.

## Conflict of Interest Statement

The authors declare that the research was conducted in the absence of any commercial or financial relationships that could be construed as a potential conflict of interest.

## Footnotes

1 MPA coders also track two other major measures. Decision Loading/Dynamism is the number of simultaneous novel or non-routine cycles of decision making an individual initiates and continues. This is measured by the presence of two or more movement effort or shape qualities in a single PGM phrase. Identifying is the spontaneous readiness to respond, participate, and become involved in an action indicated by the amount of integrated effort or shape flow movement in an individual’s overall pattern. These additional measures are among the MPA model factors that go into measuring leadership charisma in terms of motivation and style.
